# Danish feasibility study of a new innovation for treating alcohol disorders in primary care: the 15-method

**DOI:** 10.1186/s12875-022-01639-5

**Published:** 2022-02-28

**Authors:** Peter Næsborg Schøler, Jens Søndergaard, Sverre Barfod, Anette Søgaard Nielsen

**Affiliations:** 1 The Unit for Clinical Alcohol Research, Department of Clinical Research, University of Southern Denmark, Odense, Denmark; 2 The Research Unit of General Practice, Department of Public Health, University of Southern Denmark, Odense, Denmark; 3 Retired general practitioner, former researcher in alcohol topics in primary health care, Frederiksværk, Denmark

## Abstract

**Background:**

The 15-method: a new brief intervention tool for alcohol problems in primary care has shown promising results in Sweden for mild to moderate alcohol use disorders. The present study evaluated the 15-method’s usability, organizational integration, and overall implementation feasibility in Danish general practice in preparation for a large-scale evaluation of the method’s effectiveness in identifying and treating alcohol problems in general practice.

**Methods:**

Five general practices in the Central and Southern Region of Denmark participated: seven general practitioners (GPs), eight nurses. Participants received a half day of training in the 15-method. Testing of implementation strategies and overall applicability ran for 2 months. A focus group interview and two individual interviews with participating GPs along with five individual patient interviews concluded the study period.

**Results:**

Results indicate that implementation of the 15-method is feasible in Danish general practice. The healthcare professionals and patients were positive about the method and its possibilities. The method was considered a new patient centered treatment offer and provided structure to a challenging topic. An interdisciplinary approach was much welcomed. Results indicate that the method is ready for large scale evaluation.

**Conclusions:**

Implementation of the 15-method is considered feasible in Danish general practice and large-scale evaluation is currently being planned.

## Introduction

Alcohol has severe impact on public health worldwide and in Denmark; one in six Danes exceeds the nationally recommended risk level for weekly alcohol consumption [1] and more than 5% of yearly deaths are related to alcohol [2]. In Denmark, only one in eight with alcohol use disorder (AUD) and hardly anybody with less severe, but still unhealthy alcohol use, seeks treatment in specialized treatment institutions [3]. In fact, a person with unhealthy alcohol habits or milder AUD is far less likely to receive specialized treatment, than a person with severe dependence [4, 5]. There may be several reasons for this and the barriers for seeking specialized treatment are many [6, 7]. As a large part of the Danish population exceeds the recommended risk levels, large public health benefits are to be gained if alternative platforms for identifying and treating unhealthy alcohol habits and alcohol problems are developed [8–10]. A continuous challenge is how to identify and reach persons with mild and moderate AUD [11, 12], but primary care and in particular general practitioners (GPs) are considered the most relevant and promising platform and professionals to involve.

General practice is a very opportune place to both identify and treat alcohol related problems and AUD [13] as the majority of the population in the western world is in contact with their GP every year [14]. Many health related topics addressed in general practice can be affected by alcohol [15, 16]. The GP is a credited adviser regarding health and lifestyle [16] and the majority of adults find it acceptable to be asked about alcohol habits and most believe the healthcare professional (HP) should do so [17, 18]. The structure of the Danish health care system offers a continuity where such inquiries can be made in an established relation, as every citizen in DK is listed with a specific GP [19]. This ensures coherent communication to the general healthcare system, but also ensures the possibility of a closer relation to the patient and more thorough knowledge of the patient’s overall well-being. Danish general practice currently handles alcohol problems ranging from exceedance of national recommendations to harmful use and milder dependence [20].

In spite of the potential, the identification of alcohol problems among patients in primary care has so far proven difficult [21, 22]. Despite substantial effort, the implementation of treatment options for alcohol disorders in primary care continues to be a challenge [23, 24]. However, a recent innovation of screening and brief intervention (SBI) for alcohol problems in primary care has been presented: the 15-method [25, 26]. The 15-method is designed as an opportunistic screening tool and treatment option for alcohol problems ranging from hazardous use to alcohol dependence using a stepped-care approach [27].

The 15-method is an easily learned and easily implemented method to help GPs to address and treat individuals with AUD. The 15-method was developed and has been tested in Sweden in a population similar to the Danish. The trial by Wallhed, Hammarberg, Andreasson (2018) [25] was conducted on a randomized population and compared the 15-method in general practice to specialist treatment and found the 15-method was non-inferior to specialist treatment for patients with mild and moderate AUD. This group of patients had a 39% reduction in alcohol consumption and reduced their drinking problems and severity of dependence at six and 12 months follow-up [28]. The 15-method protocol has been described elsewhere [11, 23, 25].

In order to investigate if the 15-method is feasibly in the Danish structure of primary care and regarded useful by Danish GPs and patients, a Danish feasibility study of the 15-method was conducted.

The aim of the present study was to assess the feasibility of the 15-method in a Danish Primary care setting, and thus to: 1) investigate how the 15-method, including the supportive material developed in Sweden and translated into Danish, was perceived by Danish GPs, 2) investigate if and how the 15-method could be integrated in daily routines in Danish general practices. Lastly, 3) to assess if aspects of the 15-method, or the implementation of the 15-method, should be adjusted before testing the effectiveness of the 15-method in a larger scale in Denmark.

## Methods

### Study design and epistemology

The present study is a qualitative evaluation of the feasibility of the 15-method in Danish primary care. The 15-method’s feasibility is evaluated based on qualitative interviews with patients and healthcare professionals.

The feasibility study protocol is reported in accordance to CONSORT guidelines for pilot and feasibility trials [29]. The present study follows the COREQ 32-item checklist for reporting on qualitative studies [30].

The present study is based on individual and focus group interviews and uses a deductive approach [31]. We adopted an essentialist/realist position for analysis and reporting as our goal is substantive explicit evaluations from the participants and an explicit description of their experiences and meanings [32, 33]. The methodological framework and analytic procedure are described in detail in data analysis.

### Research team, reflexivity, and relationships

The research team includes: Peter Næsborg Schøler (PNS), medical doctor, research assistant; Anette Søgaard Nielsen (ASN), professor, PhD, and Jens Søndergaard (JS), professor, MD, PhD, general practitioner, clinical pharmacologist. Initiation of the study was aided by SB, general practitioner. ASN and SB have thorough experience in the research field of alcohol and treatment of alcohol problems and JS in the field of general practice research. ASN and JS have extensive experience in qualitative analysis. PNS, JS, and SB all have experience from working in general practice while ASN has extensive experience from organizing alcohol treatment on municipally level. The combination of research and clinical experience in the research team allows for analysis of study data from several angles, from a hands-on pragmatic focus in the clinics to an organizational level of multisite implementation challenges.

JS, SB, and ASN all have a solid theoretical preconception of motivational interviewing as they all have been trained themselves, and have trained others, in the approach.

PNS initiated all contact to the practices for recruitment to the study and training in the 15-method but had no prior relationship to any of the participating healthcare professionals (HPs). JS had prior to study commencement professional relationships to several of the GPs through his work as head of the Research Unit of General Practice, Odense, but did only part take in recruitment strategy and the training of GPs and not in the intervention itself or in the interviews. ANS had no prior relationship to any of the HPs. Only PNS had contact to patients for concluding interviews and there were no prior relationships between the research team and the patients. The research team was blinded from the patients’ GP affiliations. Interviewer characteristics, such as credentials and interest in the topic, was reported to all interview participants by interview initiation.

### Participants

#### Settings and location

The feasibility study was conducted in general practices in the Regions of Southern and Central Denmark. General practice in Denmark bears much resemblance to the concept of family practice and serves as a gatekeeper in the primary healthcare system. The GPs are self-employed and work on a nationally negotiated contract between GPs and the government. Healthcare services are free for the patients, as in hospital services. On average a practice holds two GPs, nurses, and secretaries. A listing system ensures every Danish citizen is listed with a specific GP. On average a GP has 1600 patients affiliated (i.e. listed) [19].

#### Eligibility and allocation

No formal eligibility criteria were set for participating practices or patients. Allocation of participating practices was not applicable. No patient randomization was possible as the 15-method’s opportunistic screening approach is based on the patients’ reasons for contact and/or symptoms. The use of the approach for potential alcohol related problems was, thus, based on the healthcare providers formal training and use of the method. As a result of this, no formal in- or exclusion criteria could be set.

#### Recruitment of general practices

General practices were contacted via e-mail and phone. All invited clinics were informed of the study’s scope and duration and briefly informed on the concept of the 15-method. Two clinics received an in-person information visit by PNS. Follow up on participating practices was made via e-mail and phone.

The intended number of participating GPs prior to recruitment was 10.

### Data collection

Two interview guides were developed: one for the HPs and one for the patient interviews. The interview guides were developed by PNS and edited by ASN and JS. The interview technique was semi-structured with questions ranging from detail oriented to general reflections on the theme of alcohol and health, while including prompts to stimulate ideas and discussion. No repeat interviews were carried out.

Interviews with the HPs were conducted via video for both individual and focus group interviews.

The participating GPs were encouraged to supply patients, who had in some way been involved in the study, with a card giving the patient the opportunity to anonymously supply the research team with contact information if they wished to participate in concluding interviews.

The intended number of patient interviews prior to intervention start was five. All interviews were audio-recorded for subsequent transcription. The duration of the individual GP interviews were approximately 60 min. The focus group interview had a duration of 90 min while all patient interviews had a duration of 30 min.

Interviews with the HPs were conducted by the end of the intervention period. Patient interviews were conducted with a planned time gap of 3 months to the conclusion of the intervention period. This allowed for follow-up consultations and evaluation of the method during three to 4 months of use. All patient interviews were conducted via phone during February and March 2021.

Storage of recordings and analysis of data were carried out on the secure Odense Patient data Explorative Network (OPEN) [34] platform in compliance with the European General Data Protection Regulations.

### Intervention: the 15-method

The 15-method [11] is an evidence-based Stepped Care approach [27] for alcohol problems. It is developed for general practice as a Screening and Brief Intervention (SBI) tool [24]. The method combines elements from Motivational Interviewing (MI), Cognitive Behavioral Therapy (CBT), bibliotherapy and pharmacological treatment. The method contains three steps, initiated by an opportunistic screening. This makes the patients’ symptoms or concrete issues the starting point of the first step. If potential problems are identified, the Alcohol Use Disorder Identification Test (AUDIT) [35] is offered for an objective assessment of the alcohol use.

Step one is to raise the topic, recognize symptoms or complaints that could be alcohol-related and give brief advice [36]. If step one is not sufficient, Step two offers a thorough assessment of the situation with feedback [37]. If the patient is willing to receive a short treatment course aimed at reducing the alcohol intake, Step three is initiated. Step 3 consists of four sessions of guided self-change treatment [38, 39] which can be combined with pharmacological treatment. The name of the method refers to the duration of each session, 15 min, and to the intended patients: those with an AUDIT score of > 15 point.

Additional information and material on the method can be found on the developers’ website [40, 41].

### Changes in intervention procedures

In the Danish feasibility study of the 15-method, minor changes were made to the original 15-method protocol [11, 25] as presented by Wallhed, Hammarberg et al. This was done to accommodate for national guidelines and organization of the Danish GPs:

1. Blood sampling procedure did not include carbohydrate-deficient transferrin (CDT) nor Phosphatidylethanol (PEth) as these blood tests are not currently included in Danish guidelines [42, 43].
2. The training session of healthcare staff in 15-method manual was reduced to two-and-a-half hour. The shortening of the training session was made with consideration to the GPs’ time schedule after dialog with the Committee of Multipractice Studies in General Practice, a part of the Danish College of General Practitioners.
3. The training was conducted as a hybrid session with the possibility of online participation due to COVID-19 restrictions.
4. The second step of the 15-method (feedback) was divided into two consultations with a duration of 15 min each, as the 30-min duration of the original feedback session was not applicable in most Danish general practices.

Two additional changes were made to facilitate implementation:

1. In the feasibility study protocol, all healthcare staff (GPs, nurses, assistants) within the practices were invited to the training session and an interdisciplinary use of the 15-method within the practices was encouraged (in the original protocol, only GPs participated in the training sessions).
2. Halfway through the intervention period an online session was held for support, Q&A session and sharing of experiences between participating GPs.

### Training and implementation

The training session of HPs had a duration of two-and-a-half hour and included: an overview of the topic of alcohol in regard to alcohol-related morbidity, mortality, and reasons for contact to the health care system. A walk-through of the 15-method material with examples of use and discussion of relevant patient groups and consultations. Video examples of the 15-method used in consultations.

The HPs were compensated for their participation in the training session, the interviews and further for 1 h of reading and getting familiar with the material.

The practices implemented the 15-method the week following the training session. It was encouraged to test different interdisciplinary approaches to the method and to test the method in different types of consultations (e.g., yearly controls, chronic disease controls, medicine check-ups or day-to-day consultations) as they found it most beneficial.

### Data analysis

Thematic content analysis was conducted within a realist methodological framework [33]**.** The analysis was theoretical as it was driven explicitly by the analyst from specific research questions, in contrast to questions evolving during the coding (i.e., an inductive approach). A theme in thematic content analysis represents both patterned responses and meanings which captures something important to the research questions, but is not dependent on quantifiable measures [33]. In line with the theoretical approach, the identification of themes were done on a semantic level with grouping of explicit meanings and statements [44]. This implies, that a unidirectional relationship is assumed between the statements of the participants and their meaning and/or motivations [45, 46]. The semantic patterns, or themes, were thus summarized based on their surface meaning and the interpretation of these themes were done focusing both on their explicit meaning but also on their implications and broader importance [47].

All data was transcribed verbatim by PNS. All transcribed data was read in full by ASN and JS prior to coding. Coding was conducted by PNS, using a concept map encompassing four prespecified topics relating to the overall research questions. Codes were then grouped into semantic themes and organized according to the overall topics during the analysis process. PNS, ASN and JS discussed the thematized data until consensus was reached on the structure and organization of the themes. Data was deemed saturated during the analytic process by PNS, ASN and JS [48].

Triangulation was conducted using disparate data sources (in-depth individual interviews with GPs, focus group interviews with HPs and individual patient interviews) and by analysis of data by different researchers [31].

All analysis was conducted using Nvivo 1.3 software.

### Participants and recruitment

**Fig. 1:**
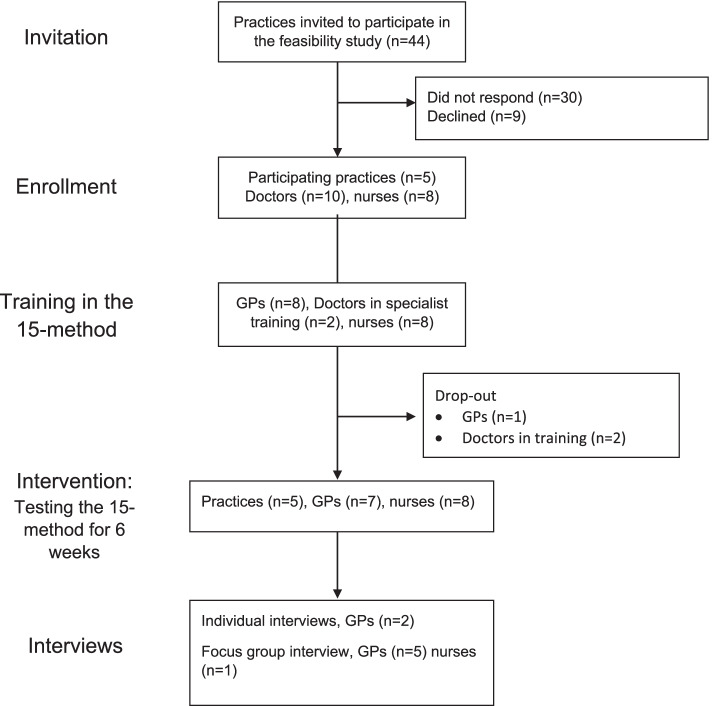
Flow chart of participating practices and healthcare professionals

A flow chart of participating practices and healthcare professionals is presented in Fig. 1.

Information on interview participants is presented in Table 1.

**Table 1:** Interview participants

**Participating healthcare professionals**				
Participant	Clinic	Position in clinic	Gender	Type of interview
1	B	General practitioner	Male	Individual
2	A	General practitioner	Female	Focus group
3	A	General practitioner	Male	Focus group
4	D	General practitioner	Female	Focus group
5	A	General practitioner	Female	Focus group
6	C	General practitioner	Male	Individual
7	E	General practitioner	Male	Focus group
8	E	Nurse	Female	Focus group
**Participating patients**				
Participant	Affiliation to practices unknown		Gender	Type of interview
1	–		Male	Individual
2	–		Male	Individual
3	–		Male	Individual
4	–		Female	Individual
5	–		Female	Individual

#### Healthcare professionals

The recruitment period of participating practices occurred from April to September 2020.

Duration of the intervention period (i.e., testing of the 15-method) was 6 weeks starting November 2nd 2020.

The practices who were invited to participate were randomly selected on a map of the region in which the study was designed to take place (The Region of Southern Denmark). An equal distribution of invited practices throughout the region was strived for, with an equal amount of rural and city-based practices. The random selection on the map was conducted by PNS, who had prior knowledge on one of the practices through his own work in the practice. All invited practices received a phone call and an e-mail with the invitation.

Five of 44 invited practices agreed to participate. 30 clinics did not respond to invitations by e-mail and phone and nine declined the invitation. The main reasons for declining were lack of time and new protocols or increased work pressure due to the COVID-19 pandemic. The number of participating HPs in total were seven GPs and eight nurses.

For the evaluation interviews two GPs were interviewed individually while the remaining five GPs and one nurse participated in the focus group interview. Healthcare interviews were conducted in December 2020.

#### Drop-out

The initial number of participating doctors (GPs and doctors in specialist training) was 10. Prior to intervention start, one GP opted out due to logistical challenges, while two practices’ doctors in specialist training opted out before the intervention period.

#### Training sessions

Introduction to and training in the 15-method was conducted prior to the intervention period. Five GPs and two doctors in specialist training participated in person. One GP participated via video while two GPs were not able to participate in the training session. The GPs who did not participate in the training session received a phone call and/or an in-person visit to the practice by PNS for information and training.

Two nurses participated in the training session. Six nurses, who did not participate in the session, were following the session trained and supervised in the method by the GP from their practice.

### Patients

The HPs were not instructed to register the number of consultations in which they made use of the 15-method or inquired about alcohol habits, as the focus was trying out the method, the material, and the implementation strategies. As a result of this, no exact estimate of the number of patients involved with the 15-method during the intervention period can be made. As the implementation strategy of the 15-method varied between practices, the estimated number of patients involved in any degree with the method varied considerably. This is illustrated by the following two examples: one GP estimated having used the method with five patients, while one practice (one nurse, one GP) implemented the method into all consultations regarding lifestyle check-ups and chronic disease and/or medicine controls and estimated the opportunistic screening approach had been applied in more than 100 consultations during the 6 weeks.

## Results

### Healthcare professionals

A total of four overall themes were identified in the HP interviews: 1) The 15-method as a new tool, 2) The material and structure of the 15-method, 3) Training and support and 4) Organization and implementation. Within the first theme, a subset of two minor themes where identified: “Relationship between patient and HP” and “Challenges”.

#### The 15-method as a new tool

Overall, the HPs felt the 15-method was a help for them in their clinical work and it served as a new tool:

It has definitely been a help, yes. I think it is a good tool … (GP1)

The participating HPs all agreed their focus on alcohol was increased by participating in the study, and several of the participants noticed how having the 15-method as an option not only increased their awareness of potential alcohol problems, but also changed how they addressed problematic alcohol use among the patients:

… because I experienced that the material changed my behavior … previously I might have been more prone to talk pharmacological options at a very early stage. But we actually postponed it and said: let’s try and register, go ahead and set some goals, fill this in – and so forth … before we came to the medication … and he [the patient] actually ended up not getting any medication and he had most likely been equipped with some sort of pharmacological treatment – Campral – who knows, before this material … and I find that somewhat interesting (GP 4)

Most of the HPs found the approach of the method made it easier to address and talk about alcohol habits, mostly because the approach offered a structure for the conversation and had clinical issues or concrete problems as its reference point throughout the process.

[Yes] well, this gives a structure to how one can approach it [the topic of alcohol], in a very tangible way (GP 1).

In particular, the HPs stressed that the 15-method filled a gap in their ‘tool-box’: the method provided them with something to offer a patient group that otherwise was considered difficult to reach. It served both as an in-between step in the treatment approach within the practices, e.g., pharmacological treatment, but also between the existing treatment offers in the institutions surrounding general practice (e.g., municipally treatment options). As such, the 15-method served as a potentially missing link in the current options both within the practices and regarding referrals.

… it is a new treatment offer for the patients we have here, I believe that it is … I think it makes a lot of sense to be able to offer this option (GP 6).

Identification of alcohol problems and the relationship between patient and healthcare provider.

The opportunistic approach of the 15-method was overall evaluated as useful. Although most of the HPs still experienced patients who were not willing to talk about their alcohol habits, four of the five practices had become more aware of alcohol problems among the patients, addressed them more often and found it easier to do so:

We find in some way it has been a barrier for us before. But we have experienced it is pretty easy to get going asking people about their alcohol habits (GP 1).

The use of screening tools, i.e., the AUDIT questionnaire, worked very well when presented in the right context (opportunistic) and was in several cases an eye-opener for both patients and GPs:

… I think the questionnaires are really great and relevant. And they have worked really well. The initiating one [AUDIT] and the screening is really great for, like, opening your eyes and see if there really is a problem, because in this way it is not me sitting there, judging, and saying: “where are you placed on the colored scale?” – it’s the patient himself who, from his answer, can say: “Whoops! I’m placed there!” – That works really, really well (GP 4).

We have actually been surprised by some [patients], who we didn’t expect to drink that much – who really drank a lot (GP 1)

Although the 15-method, including the screening tools, was considered suitable for a range of patients with alcohol related problems by the HPs, the HPs also noted limitations. It was, for instance, emphasized that patients of limited resources struggled with e.g., the amount of reading necessary in the material and filling out questionnaires and that patients struggling with several other issues would be quicker to decline conversation regarding alcohol habits:

… it is the more resourceful [patients] who can participate in this … perhaps they could fill in the AUDIT with some help, but … we have patients with limited resources … if one was helped at home … it’s less stigmatizing to get help measuring your blood pressure, than having your kids filling out your alcohol habits … who do you want to help you with that? (GP 6)

Three HPs expressed a need for becoming more familiar with the treatment protocol in order to become able to act more freely in the conversation and decision making with the patients. Familiarity with the material and nature of the relationship to the patient was found by some to be of importance when deciding whether or not to screen for and address problematic alcohol use. Others, however, felt that the screening process and use of the material (questionnaires, homework assignments) was not particularly affected by their relation to the patient or in-depth familiarity with the material:

… with something like this, just as [nurse 1] says, it’s about trust. If [nurse 1] has a good relation to someone and they like talking with her, then she’s the one going forward with this … (GP 5)

I don’t think it [asking about alcohol habits or using AUDIT] is transgressive and I don’t think it requires a lot of trust – because the patients are going to answer on their own, and they might lie about it, but if they do they weren’t ready anyway [for treatment] (GP 4)

Despite these differences it was emphasized by all the participating HPs that the opportunistic screening process should originate from a plausible, grounded indication and not upset a natural agenda of a consultation, and that it was a strength of the 15-method that it was designed as a pragmatic screening and intervention tool.

##### Challenges

The COVID-19 situation posed a challenge to the practices throughout the project period, as all participating practices implemented government issued protocols regarding e.g., physical consultations and cross-sectoral priorities. Most of the HPs agreed, that physical consultations were best suited for the method, as it in most cases requires a certain degree of non-verbal communication to address and discuss a potentially sensitive topic, such as alcohol habits. Some of the GPs described how their general health check-ups and routine consultations had been downgraded to a minimum to make sure the necessary capacity in the practice was available during the COVID situation. This did affect the HPs possibilities of conducting opportunistic screening and follow up in person.

#### The material and structure of the 15-method

All the GPs agreed the overall idea and concept of the 15-method made a lot of sense and was easily comprehensible when learned. However, all participants felt the educational material for healthcare providers and the patient material was too vast and could potentially become a barrier:

I think the material [is] unnecessarily comprehensive and I would say that I think it [the concept] is simpler than it is made out to be. (GP 4)

All the GPs agreed a shorter format of the material could aid them in the everyday clinical work. Furthermore, the potential for an electronic version of most of the material was stressed since all participating general practices already made use of electronic questionnaires and patient assignments prior to consultations. The possibility of adding the 15-method to such an online bouquet to the extent possible, was a major part of the theme.

#### Training and support

The HPs considered that training in the 15-method requires a face-to-face session (as opposed to online). However, the different professions varied in how much training time they considered necessary. The GPs all had a sense that an overall introduction and managing style of training in the method was sufficient, while healthcare staff, e.g., nurses, in the clinic considered they would benefit more from a full-day workshop with a more hands-on training.

The structure of the 15-method was overall viewed as straight forward and making a lot of sense, albeit preferably requiring some prior knowledge of the concepts used in the method, such as MI.

#### Organization and implementation

The organization of the 15-method was handled differently within the practices, but as a common thread it was found, that the practices valued an inter-disciplinary approach with different roles for different professions. The interdisciplinary approach was highlighted as a strength both in regard to identification of alcohol problems, patient continuity and time-efficiency. The flexibility of the implementation was emphasized as a strength, but it was also found that it was important to keep in mind the different capabilities of the professions within the practice if the method was to be a success. Often was patient continuity and well-established relations with the nurses emphasized, with a more supervising role for the GP.

To make the screening-part something we did together, to sort of get people motivated. And then the rest of the process would be with the one in the clinic who was most familiar with the method (GP 3)

The participating practices where overall in agreement that the details of the implementation should be up to the individual practice. All GPs agreed neither the screening nor the intervention part had to be by a doctor but could be optimized by being conducted with e.g., a nurse, with the GP in a supervising role.

### Patients

**Fig. 2:**
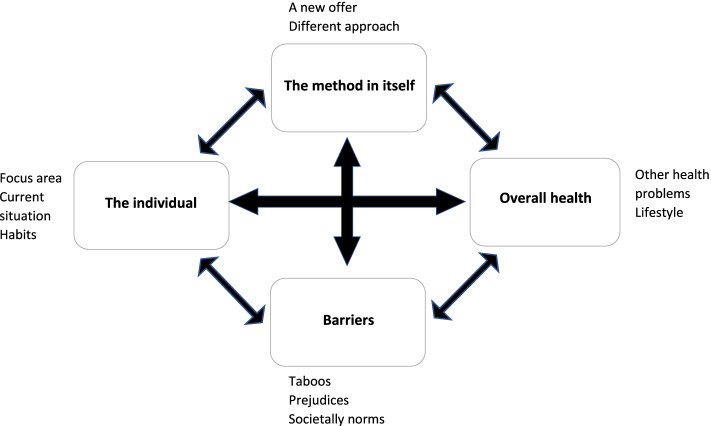
Themes and their relations identified from patient interviews

Not only the HPs, but also the patients, expressed a high level of satisfaction with the 15-method. The analysis of the patient interviews identified four major themes: 1) The individual, 2) Overall health, 3) Barriers and 4) The method in itself. Each major theme held one to three minor themes. The identified themes from the patient interviews and their relations are illustrated in Fig. 2.

#### The individual

The patients, who were interviewed about their experience of the 15-method, described how the conversation with the HP made them reflect on their alcohol use.

[ … ] where before, I probably hadn’t thought of it as being a problem [ … ] and if I were to look at it [alcohol consumption] – closely – then it adds up … I actually think I got quite surprised. Because I hadn’t thought of it like this before. Like, that’s one, that’s two, that’s three drinks. I hadn’t thought of how many drinks it actually amounts to. That was not until I was actually writing it down. And that actually made me a lot more conscious – I have become much more conscious [about my alcohol consumption] (Patient 5)

The patients’ view on own health, own situation and own lifestyle habits affected how they viewed the 15-method to fit into their life and to which extend they felt the intervention to be helpful. In each case, regardless of the duration of the 15-method intervention they were offered, the conversation with the HP affected the patients’ reflections on their alcohol habits and in most cases, it affected the alcohol habits themselves.

The patients’ relationship to their healthcare provider influenced the process in different ways. The patients were inclined to prefer that the conversation about alcohol was initiated (e.g., the screening part of the intervention) and conducted (e.g., the short treatment sessions) by a nurse rather than the GP him or herself. The main reasons for this was a feeling of having more time and feeling closer to the nurse, whom the patients tended to see more often than the GP. An element of authority, however, also appeared, as one of the male patients described how he preferred the GPs guidance, while the female patients appreciated the more relaxed and intimate mood of consultations with a nurse. As such, preferences for which HP provided the guidance was, potentially, influenced by gender differences.

However, it is important to notice, that not all patients felt the familiarity with the staff was an advantage. One patient described how he would rather talk about his alcohol habits with an HP outside the practice (e.g., a psychologist), as he felt the familiarity inhibited him from being straightforward.

#### Overall health

How the patients experienced the 15 method was influenced by their overall health. Simply discussing alcohol habits with the HP was considered transgressive by the patients if alcohol use was discussed as an isolated issue and not in relation to health or specific symptoms. However, all patient noted alcohol was a legitimate and necessary topic to raise if the alcohol use was tied to a specific health problem, seen as part of their own problem in a broader sense or discussed as a direct aspect of their well-being or health problem. Thus, all the patients agreed that alcohol was a natural part of, or should be a natural part of, conversations in order to give a holistic picture of their health, even though the topic at first might seem uncomfortable or transgressive. Two of the patients described how they were moved from feeling the topic as being transgressive to becoming acceptable and even helpful during the conversation. As such, no patient deemed the overall experience to be transgressive, when seen in a broader perspective, as illustrated by the following example:

I: Did you find it transgressive, for this topic to be addressed?

P5: [um] yes, of course it was. It’s never a whole lot of fun to talk about one’s lifestyle problems. I’m overweight, I eat wrong, don’t exercise enough and drink perhaps a bit too much alcohol. In some sense it’s embarrassing and somewhat of a defeat to talk about, so [yeah] – it’s never pleasant to be reminded that one should alter habits.

I: … but had you, before this came up, thought of alcohol as a problem – or that it could be a problem?

P5: Actually, I hadn’t.

I: No?

P5: If I’m being honest

I: No. So, was it okay as an eye-opener, or how did it make you feel? [ … ]

P5: Well, it did help make me more conscious about it. So [yeah], I think it has been just fine. Absolutely.

#### Barriers

All patients found that alcohol played a part in many aspects of their social life and spare time and was a much integrated part of their life. As most of the patients started reflecting more on their alcohol habits as a result of it being addressed in the practices, it affected their view on alcohol in relation to societal norms and their peers. How the patients talked about alcohol with their family and friends varied much, as did the concept of whether an alcohol “problem” was present. This latter reflection affected both how the patients saw themselves in relation to alcohol, after the topic had been addressed, and their thoughts on alcohol as part of their everyday life. Most of the patients found it challenging to alter their alcohol habits in social contexts. This was due to both social norms and taboos. These topics encompassed how the patients spoke about alcohol with their network, if at all, and taboos related to seeking treatment and addressing potential excessive alcohol use among friends or family.

It’s just not considered a problem. I mean, many in our circle of friends share a bottle of wine or two on a given evening, and it’s not considered problematic, you know? [ … ] but if you where to look at the recommendations it would actually be considered [excessive] use [ … ] We only see it when people become bad-mannered. When they get drunk or if they can’t take care of their job. [ … ] Or if it has other consequences [ … ] but we don’t look at the effect it has on ourselves, because no one else feels that – just us – as an individual [ … ] but that’s just as important. (Patient 4)

There is no doubt, that a lot of people – like me – drink too much without really noticing it [ … ] because it’s just a part of everyday life, you know? [ … ] one drink here or there [ … ] you don’t see yourself as the drunk sitting in the bar or down in the park every day [ … ] it’s much more hidden than that. It’s at home, behind the four walls of your house, together with someone you feel safe with. That makes it less dangerous. It makes you not think of it as a problem – but it is! It really is! (Patient 5)

#### The method in itself

Most of the patients viewed the 15-method as a new offer with a different approach to their current situation. It was found that the method for some patients filled an important potential gap in the current offers in the health care system, as they would not have gone elsewhere for treatment:

P4: I have been online [ … ] to look at … well, to look up alcohol abuse, right.

I: Yes, yes

P4: And in my head – it was either or. Either you drink or you go into rehab. And I just felt like – that’s not where I am, I think.

I: No.

P4: But I don’t think it has been easy to find help.

I: No?

P4: And it [the 15-method] really appealed to me, because I though all along that it was an either-or. [ … ] Either Antabus, zero alcohol, or keep doing what you’re doing and manage it on your own. But this is like in between, a way to minimize it.

I: Yes

P4: That really appealed to me [ … ] I think it was great.

P5: I feel safe with him [GP]. At the municipal treatment facility – I don’t feel comfortable with them at all.

I: No?

P5: I wouldn’t open up at all in such a treatment setting. With my doctor I want to be honest, because – I have to, if he is to help me with all my other problems.

I: Yes

P5: But at the treatment center – I don’t feel like I have any responsibility to tell them anything at all.

All patients agreed that the 15-method approach affected their alcohol habits in some way. It was further found that alcohol consumption was reduced during the intervention in all patient cases. One of the main ways the method influenced the patients’ habit and reflections on alcohol consumption was as an eye opener:

It wasn’t something I was conscious of, because it not like I sit down in the park with a bottle of schnaps every day [ … ] So I wasn’t aware of it before I actually started writing it down: Okay, that’s one drink, that’s another, and that’s another – okay! There are really weeks where I drink too much! (Patient 5)

The patients found the method well tied into a holistic perspective of their health and lifestyle. The focus on general health, with the method as a piece of the puzzle, was appealing to the patients. All patients found the topic of alcohol important, both in general and to their own health. They found it equally important to address the topic in a holistic perspective but tied to their specific situation or problem.

Patients presented with the material found the patient material to be too vast. It was emphasized by some patients, that during a time when one needs help from e.g., one’s GP, more problems can be intertwined. In such a time, patient material is best presented in small, manageable pieces. Potential improvements to the material thus included condensation and separation into smaller items.

Main findings from healthcare and patient interviews can be found in Table 2.

**Table 2:** Main findings from healthcare and patient interviews

Healthcare professionals	Overall, the 15-method was considered to being a helpful tool:
	To initiate the conversation about alcohol habits.
	In giving structure to a challenging treatment area.
	As a flexible and individualizable option for the patients.
	The method was found to have the potential of a new treatment offer for the targeted patient group.
	The method was found to work well in an interdisciplinary approach. The healthcare material should be condensed.
	The material should be digitalized to the broadest extent possible.
	The implementation of the method needs adjustment (e.g., time spent on each step, organization of responsibilities in the practice)
Patients	The 15-method felt as a personalized approach.
	The approach was perceived as having the potential to being a natural part of a bigger perspective on health and lifestyle.
	The method was found to be a much-welcomed new treatment option (i.e., treatment goals, holistic perspective, logistics, less stigmatized).
	The patient material should be condensed and divided into smaller pieces.
	The material should be digitalized.

## Discussion

The present study is an evaluation of a feasibility study of the 15-method in Danish general practice. Evaluation included how the method was perceived by Danish GPs, how the method could be integrated into daily routines and which aspects of the method and implementation procedures to adjust before large scale testing of the method.

Overall, the 15-method was viewed as a helpful tool, providing structure to a topic generally considered challenging to approach. The 15-method offered an option residing between existing treatment options in the Danish healthcare system, for patients and HPs in general practice. Thus, the 15-method might potentially fill a gap in the Danish health care system between current available treatment offers for alcohol problems. Those of general practice and municipal treatment facilities.

The interdisciplinary approach was found beneficial, something already emphasized as a way to improve the 15-method [26].

The material for HPs and patients was considered by the participants in the present study as being too vast and too ‘pen-and paper’-based. It was suggested to digitalize the material, questionnaires, and tools in order to ease the integration into daily clinical practice. This is important knowledge to take into consideration before a larger scale implementation of the 15-method in order to test its efficacy in Danish primary care. Digitalization and integration into the electronic patient records system may allow for development of automatic reminders to the HP about raising the topic of alcohol in relevant situations and thus aid the implementation process.

The flexibility of the integration into the practices and relatively short training sessions were considered valuable for the overall implementation process.

Implementation of screening and brief intervention protocols for alcohol problems have been a challenge in primary care for the past decades [23, 24], and so has bridging the gap between primary care and specialized treatment options [49, 50]. The 15-method includes aspects which can improve implementation of behavioral change interventions, such as training and education [51–55], on-the-job experience with the intervention [54] and the possibility of interdisciplinary approaches [51, 52]. The present study indicates that the 15-method offers an easily learned method that seems to make sense in general practice. In particular, it is positively perceived due to linking the symptoms and health of the patients to potential excessive alcohol use. Furthermore, the participants in the present study found the 15-method offering a strategy for how to perform a non-confrontative conversation with the patient about alcohol, and a strategy for how to treat alcohol problems in primary care in a rather simple manner. Thereby, the 15-method seems to be able to overcome often stated barriers towards addressing alcohol in primary care, for instance lack of time, fear of offending patients or damaging established relations [11, 52, 56]**.** Also of considerate importance, the 15-method has shown promising results in decreasing alcohol consumption in the group of patients who is located very much in the described treatment gap [25, 28].

From the results of this feasibility study the following amendments are considered for future larger scale evaluation: The interdisciplinary approach encouraged in the Danish feasibility study of the 15-method was evaluated as a success and should be carried over to large scale evaluation.

The length of the training sessions should be adjusted to fit the healthcare profession, i.e., a shorter training session for GPs with an overall, supervisory focus, and longer hands-on workshop for practice staff, e.g., nurses.

Material for professionals and patients should be condensed and divided into smaller individual pieces to facilitate ease of use. A flow chart for the course of treatment should be developed and the method should be implemented in the practices’ patient filing systems to the broadest extent possible. The condensed version of the material will continue to follow the structure and have the content of the original 15-method, i.e., a targeted screening and stepped-care approach, with the use of supporting material as needed (e.g., AUDIT). AUDIT and the additional screening instruments included in the 15-method will thus continue to be supportive tools for the clinicians to be used when deemed relevant.

Overall, the 15-method was well received by the patients and overall considered a new treatment option. Although the patients interviewed within the present study were few, it is notable that they were positive towards receiving interventions in primary care, aimed at reduction of alcohol intake. In general, alcohol intake is considered an important part of Danish daily life and associated with quality of life. The patient angle in the present study described how the patients’ perception of alcohol use was affected by the 15-method-conversations with the HP and increased awareness towards how alcohol affected their health. Also, despite that alcohol is considered a sensitive topic, it was of importance to learn that the patients found it meaningful to be approached by the HPs if alcohol use might be a potential explanation for symptoms or if it might affect the patients’ health.

The present study was affected by the Covid-19 situation. The initiation of the study period, including the recruitment of practices, coincided with the outbreak of the Covid-19 pandemic and the following governmental restrictions and legislations in Denmark. As a result of this, many invited practices were overworked during the study’s recruitment phase trying to implement new government guidelines and protocols for handling the Covid-19 situation. The invitation procedure itself was designed to minimize selection bias, but many of the practices who declined stated that the unusual circumstances regarding the Covid-19 situation used up most of their capacity for other projects and protocols in the practice. The starting date of the intervention period (i.e., testing the 15-method) was thus postponed. This resulted in the intervention period being shorter than initially planned. The shortening of the intervention period limited the number of possible consultations, hands-on time, and experiences with the 15-method. Further, the unusual circumstances during the recruitment period might have kept some practices, who would otherwise have accepted the invitation, from accepting the invitation to participate.

Secondly, the feasibility study was conducted in the Danish primary health care, which in several ways is like the Swedish, where the 15-method was developed. The evaluation from a Danish healthcare perspective entails a limited generalizability to health care systems outside of Scandinavia.

Also, two important potential biases in the evaluation of the intervention must be considered. First, the present feasibility study, and subsequent evaluation, was completed by general practices positive towards the project. Furthermore, they could be considered frontrunners amongst their peers, as they are actively involved in other research projects. Second, only patients who agreed to discuss alcohol with their HP and agreed to be contacted by the research team, were interviewed.

It is thus reasonable to assume that the HPs participating in the present study were overall more positive towards testing new interventions and likewise, that the patients were more positive towards talking about alcohol with their HP, as they did not decline the offer. Further, it could be argued, that the patients found it less transgressive overall to discuss the topic of alcohol, as they agreed to be interviewed on the topic. These factors could lead to an overall more optimistic evaluation of the intervention.

We acknowledge, that the participating practices were a selected group and interpretations of the results were made in light of this. The views of non-responders have not been explored in this study. To address this issue, the exploration of current views among non-responders has been included in a planned future study investigating the barriers and facilitators for addressing alcohol in Danish general practice.

Another limiting factor is the relatively small sample of interviewed patients and HPs. However, data was deemed saturated by the research team during analysis.

It may be considered a strength of the present study that the 15-method was implemented in daily routine and that flexibility in how to implement it was encouraged. Thus, the feasibility study provided new knowledge of importance to future effectiveness testing and evaluation of the method in the Danish primary health care.

## Conclusions

The 15-method offers a warranted approach and treatment for alcohol problems in general practice. The 15-method could potentially help fill a treatment gap within current treatment options in Danish primary health care. This indicate that implementation of the 15-method is feasible in Danish general practice. Large scale testing of the 15-method’s effectiveness is needed to evaluate if the method is recommendable for countrywide dissemination and implementation.

### Planned future studies

The larger scale effectiveness testing and evaluation of the 15-method in Danish general practice is planned. Following the present feasibility study, an effectiveness study will be carried out in a stepped-wedge cluster randomized control trial. The effectiveness study will include quantitative analyses of the 15-method’s effectiveness in increasing the proportion of alcohol related conversations in the participating general practices. Further, the study will analyze the 15-method’s effectiveness in lowering the proportion of persons who exceed the Danish nationally recommended low-risk alcohol consumption level, among patient affiliated with the participating practices. Additional quantitative measures are included (e.g., blood samples, patient questionnaires). A process evaluation study will be carried out during the large-scale testing in a mixed-methods design.

Studies on the implementation process of the 15-method during the effectiveness study will include analyses of perceived barriers and facilitators among healthcare professionals and patients. The studies will further analyze key aspects of the implementation process, e.g., reach, acceptability, maintenance of intervention use and normalization of the workflow. These studies on the implementation process will be conducted using both qualitative and quantitative data.

## Data Availability

The dataset of transcribed interviews (in Danish) is available upon request to the corresponding author.

## References

[1] Sundhedsstyrelsen (2018). Forebyggelsespakke Alkohol. Islands Brygge 67, 2300.

[2] Sundhedsstyrelsen og Statens Serum Institut (2015). Alkoholstatistik 2015 Nationale Data. Axel Heides Gade 1, 2300.

[3] Sundhedsstyrelsen (2019). Sundhedsstyrelsens servicetjek af offentligt finansieret alkoholbehandling.

[4] Kohn R, Saxena S, Levav I, Saraceno B (2004). The treatment gap in mental health care. Bull World Health Organ.

[5] Storbjörk J, Room R (2008). The two worlds of alcohol problems: who is in treatment and who is not?. Addict Res Theory.

[6] May C, Nielsen AS, Bilberg R (2019). Barriers to treatment for alcohol dependence. J Drug Alcohol Res.

[7] Wallhed Finn S, Bakshi AS, Andreasson S (2014). Alcohol consumption, dependence, and treatment barriers: perceptions among nontreatment seekers with alcohol dependence. Subst Use Misuse.

[8] Eriksen L, Davidsen M, Jensen HAR, Ryd JT, Strøbæk L, White ED (2016). Sygdomsbyrden i Danmark: Risikofaktorer: Sundhedsstyrelsen.

[9] Room R, Babor T, Rehm J (2005). Alcohol and public health. Lancet.

[10] Marlatt GA, Witkiewitz K (2002). Harm reduction approaches to alcohol use: health promotion, prevention, and treatment. Addict Behav.

[11] Wallhed FS. Alcohol dependence: barriers to treatment and new approaches in primary care: Inst för folkhälsovetenskap/Dept of public health sciences. Stockholm: Karolinska Institutet; 2018.

[12] Kousgaard MB, Joensen ASK, Thorsen T (2015). The challenges of boundary spanners in supporting inter-organizational collaboration in primary care–a qualitative study of general practitioners in a new role. BMC Fam Pract.

[13] Rehm J, Manthey J, Struzzo P, Gual A, Wojnar M (2015). Who receives treatment for alcohol use disorders in the European Union? A cross-sectional representative study in primary and specialized health care. Eur Psychiatry.

[14] Anderson P. Alcohol and primary health care: WHO regional office Europe; 1996.

[15] Nutting PA (1986). Health promotion in primary medical care: problems and potential. Prev Med.

[16] Slama KJ, Redman S, Cockburn J, Sanson-Fisher RW (1989). Community views about the role of general practitioners in disease prevention. Fam Pract.

[17] O’Donnell A, Abidi L, Brown J, Karlsson N, Nilsen P, Roback K (2018). Beliefs and attitudes about addressing alcohol consumption in health care: a population survey in England. BMC Public Health.

[18] Coste S, Gimenez L, Comes A, Abdelnour X, Dupouy J, Escourrou E. Discussing alcohol use with the GP: a qualitative study. BJGP Open. 2020;4(2):bjgpopen20X101029. 10.3399/bjgpopen20X101029. PMID: 32345694; PMCID: PMC7330215. 10.3399/bjgpopen20X101029 PMC7330215 32345694

[19] Pedersen KM, Andersen JS, Søndergaard J (2012). General practice and primary health Care in Denmark. J Am Board of Fam Med.

[20] Bjerrum L, Barfod S. National Clinical Guideline on Alcohol in General Practice (in Danish), vol. 2010: Danish Health Authority, Practitioners DCoG; 2010.

[21] Aertgeerts B, Buntinx F, Ansoms S, Fevery J (2001). Screening properties of questionnaires and laboratory tests for the detection of alcohol abuse or dependence in a general practice population. Br J Gen Pract.

[22] Buchsbaum DG, Buchanan RG, Lawton MJ, Schnoll SH (1991). Alcohol consumption patterns in a primary care population. Alcohol Alcohol.

[23] Glass JE, Andréasson S, Bradley KA, Finn SW, Williams EC, Bakshi A-S, et al. Rethinking alcohol interventions in health care: a thematic meeting of the international network on brief interventions for Alcohol & Other Drugs (INEBRIA): Springer; 2017. 10.1186/s13722-017-0079-8 PMC5425968 28490342

[24] McCambridge J, Saitz R. Rethinking brief interventions for alcohol in general practice. BMJ. 2017;356. 10.1136/bmj.j116 28108452

[25] Wallhed Finn S, Hammarberg A, Andreasson S (2018). Treatment for alcohol dependence in primary care compared to outpatient specialist treatment—a randomized controlled trial. Alcohol Alcohol.

[26] Wallhed Finn S, Hammarberg A, Andreasson S, Jirwe M. Treating alcohol use disorders in primary care–a qualitative evaluation of a new innovation: the 15-method. Scand J Prim Health Care. 2021;39(1):51–9. 10.1080/02813432.2021.1882079. Epub 2021 Feb 15. PMID: 33586596; PMCID: PMC7971313. 10.1080/02813432.2021.1882079 PMC7971313 33586596

[27] Sobell MB, Sobell LC (2000). Stepped care as a heuristic approach to the treatment of alcohol problems. J Consult Clin Psychol.

[28] Wallhed Finn S, Andréasson S, Hammarberg A (2020). Treatment of alcohol dependence in primary care compared with outpatient specialist treatment: twelve-month follow-up of a randomized controlled trial, with trajectories of change. J Stud Alcohol Drugs.

[29] Eldridge SM, Chan CL, Campbell MJ, Bond CM, Hopewell S, Thabane L, et al. CONSORT 2010 statement: extension to randomised pilot and feasibility trials. BMJ. 2016;355. 10.1136/bmj.i5239 PMC5076380 27777223

[30] Tong A, Sainsbury P, Craig J (2007). Consolidated criteria for reporting qualitative research (COREQ): a 32-item checklist for interviews and focus groups. Int J Qual Health Care.

[31] Chapman A, Hadfield M, Chapman C (2015). Qualitative research in healthcare: an introduction to grounded theory using thematic analysis. J R Coll Phys Edinb.

[32] Morgan DL (2010). Reconsidering the role of interaction in analyzing and reporting focus groups. Qual Health Res.

[33] Braun V, Clarke V (2006). Using thematic analysis in psychology. Qual Res Psychol.

[34] Regions of Southern Denmark (2021). Odense Patient data Explorative Network (OPEN).

[35] Reinert DF, Allen JP (2002). The alcohol use disorders identification test (AUDIT): a review of recent research. Alcohol Clin Exp Res.

[36] O'Donnell A, Anderson P, Newbury-Birch D, Schulte B, Schmidt C, Reimer J (2014). The impact of brief alcohol interventions in primary healthcare: a systematic review of reviews. Alcohol Alcohol.

[37] Miller WR, Sovereign RG, Krege B (1988). Motivational interviewing with problem drinkers: II. The Drinker's check-up as a preventive intervention. Behav Cogn Psychother.

[38] Sobell MB, Sobell LC. Problem drinkers: guided self-change treatment: The Guilford Press; 1996.

[39] Andréasson S, Hansagi H, Österlund B. Short-term treatment for alcohol-related problems: four-session guided self-change versus one session of advice—a randomized, controlled trial. Alcohol. 2002;28(1):57–62. 10.1016/s0741-8329(02)00231-8 12377361

[40] Riddargatan 1 Region of Stockholm. 15 Metoden (Swedish) Available from: https://www.riddargatan1.se/utbildning-personal/15-metoden/.

[41] Denmark TUoS. The 15-method in Danish Primary Care [Webpage ]. [In Danish]. Available from: https://www.sdu.dk/da/15-metoden.

[42] Sundhed.dk. Alkoholproblemer 2018 [cited 2021 04.19.]. Available from: https://www.sundhed.dk/sundhedsfaglig/laegehaandbogen/psykiatri/tilstande-og-sygdomme/alkohol/alkoholproblemer/.

[43] Sundhedsstyrelsen. National Klinisk Retningslinje: Behandling af alkoholafhængighed. Report. Sundhedsstyrelsen, Sundhedsstyrelsen; 2018 November 23rd.

[44] Boyatzis RE. Transforming qualitative information: thematic analysis and code development: sage; 1998.

[45] Widdicombe S, Wooffitt R. The language of youth subcultures: social identity in action: harvester/wheatsheaf; 1995.

[46] Potter J, Wetherell M. Discourse and social psychology: beyond attitudes and behaviour: Sage; 1987.

[47] Patton MQ. Qualitative evaluation and research methods: SAGE Publications, inc; 1990.

[48] Saunders B, Sim J, Kingstone T, Baker S, Waterfield J, Bartlam B (2018). Saturation in qualitative research: exploring its conceptualization and operationalization. Qual Quant.

[49] Kamstrup-Larsen N, Broholm-Jørgensen M, Dalton SO, Larsen LB, Thomsen JL, Tolstrup JS (2019). Why do general practitioners not refer patients to behaviour-change programmes after preventive health checks? A mixed-method study. BMC Fam Pract.

[50] Vendetti J, Gmyrek A, Damon D, Singh M, McRee B, Del Boca F (2017). Screening, brief intervention and referral to treatment (SBIRT): implementation barriers, facilitators and model migration. Addiction.

[51] Nilsen P, Wåhlin S, Heather N (2011). Implementing brief interventions in health care: lessons learned from the Swedish risk drinking project. Int J Environ Res Public Health.

[52] Johnson M, Jackson R, Guillaume L, Meier P, Goyder E (2011). Barriers and facilitators to implementing screening and brief intervention for alcohol misuse: a systematic review of qualitative evidence. J Public Health (Oxf).

[53] Anderson P, Coulton S, Kaner E, Bendtsen P, Kłoda K, Reynolds J (2017). Delivery of brief interventions for heavy drinking in primary care: outcomes of the ODHIN 5-country cluster randomized trial. Ann Fam Med.

[54] Anderson P, Kaner E, Keurhorst M, Bendtsen P, Steenkiste BV, Reynolds J, et al. Attitudes and learning through practice are key to delivering brief interventions for heavy drinking in primary health care: analyses from the ODHIN five country cluster randomized factorial trial. Int J Environ Res Public Health. 2017;14(2). 10.3390/ijerph14020121 PMC5334675 28134783

[55] Nilsen P, Aalto M, Bendtsen P, Seppä K (2006). Effectiveness of strategies to implement brief alcohol intervention in primary healthcare. A systematic review. Scand J Prim Health Care.

[56] Nygaard P, Aasland OG (2011). Barriers to implementing screening and brief interventions in general practice: findings from a qualitative study in Norway. Alcohol Alcohol.

